# Comparison of machine learning approaches for enhancing Alzheimer’s disease classification

**DOI:** 10.7717/peerj.10549

**Published:** 2021-02-25

**Authors:** Qi Li, Mary Qu Yang

**Affiliations:** MidSouth Bioinformatics Center and Bioinformatics Graduate Program, University of Arkansas at Little Rock and University of Arkansas for Medical Sciences, University of Arkansas at Little Rock, Little Rock, AR, United States of America

**Keywords:** Very deep convolutional network, Deep residual network, Alzheimer’s disease, Gradient-weighted class activation mapping, MRI

## Abstract

Alzheimer’s disease (AD) is a progressive neurodegenerative disorder, accounting for nearly 60% of all dementia cases. The occurrence of the disease has been increasing rapidly in recent years. Presently about 46.8 million individuals suffer from AD worldwide. The current absence of effective treatment to reverse or stop AD progression highlights the importance of disease prevention and early diagnosis. Brain structural Magnetic Resonance Imaging (MRI) has been widely used for AD detection as it can display morphometric differences and cerebral structural changes. In this study, we built three machine learning-based MRI data classifiers to predict AD and infer the brain regions that contribute to disease development and progression. We then systematically compared the three distinct classifiers, which were constructed based on Support Vector Machine (SVM), 3D Very Deep Convolutional Network (VGGNet) and 3D Deep Residual Network (ResNet), respectively. To improve the performance of the deep learning classifiers, we applied a transfer learning strategy. The weights of a pre-trained model were transferred and adopted as the initial weights of our models. Transferring the learned features significantly reduced training time and increased network efficiency. The classification accuracy for AD subjects from elderly control subjects was 90%, 95%, and 95% for the SVM, VGGNet and ResNet classifiers, respectively. Gradient-weighted Class Activation Mapping (Grad-CAM) was employed to show discriminative regions that contributed most to the AD classification by utilizing the learned spatial information of the 3D-VGGNet and 3D-ResNet models. The resulted maps consistently highlighted several disease-associated brain regions, particularly the cerebellum which is a relatively neglected brain region in the present AD study. Overall, our comparisons suggested that the ResNet model provided the best classification performance as well as more accurate localization of disease-associated regions in the brain compared to the other two approaches.

## Introduction

Alzheimers disease (AD) is an irreversible and progressive neurodegenerative disorder that often happens to people older than 65 years, accounting for nearly 60% of all dementia cases (Singh et al., 2016). The occurrence of the disease has been increasing rapidly in recent years. Nowadays, approximately 90 million individuals suffer from AD worldwide, and it is estimated that the number of AD patients will reach 300 million by 2,050 (Zhan et al., 2015). The disease causes structural changes in the brain, thereby affecting behavior, cognition, emotions, and memory. Sensitive and specific AD biomarkers are imperative for prevention and early diagnosis. Structural MRI displays morphometric differences and cerebral structural changes and has been widely used in neuroimage analysis (Ledig et al., 2018; Matsuda, 2013; Trefler et al., 2016). MRI image analysis of AD might give a hint of which structures are involved in the development of AD as well as provide insights into long-term structural changes caused by AD.

Various machine learning techniques have been investigated in MRI analysis for AD classification. The commonly used classifiers include SVM (support vector machine), CNN (Convolutional Neural Network), and other ensemble classifiers (Liu et al., 2012). Among them, SVM and the variants have been widely used due to their relatively good accuracy and ability to deal with high-dimensional data (Alam et al., 2017; Lama et al., 2017; Ledig et al., 2018; Long et al., 2017). A region-based method was often used to select features as input to the SVM algorithm. The brains were first segmented into many anatomical regions of interest (ROIs) according to the MRI-space of each subject. Then the mean signal intensity in each of these regions from the MR images was extracted as input features. Although the ROI-based method has shown its effectiveness in AD classification, there are still some problems that cannot be ignored. The ROIs are generated by the prior hypotheses; thus, some minute abnormal changes may be ignored. Also, diseased-induced structural changes may occur at relatively large regions of the brain. Spatial information found in several voxel-grouped local regions, which has lost in the ROI-based method, should be considered to enhance the classification (Hayasaka & Laurienti, 2010).

In contrast to traditional machine learning algorithms, deep learning algorithms are able to learn the latent features of the data automatically and have been known for high accuracy in learning relevant features for classification tasks, especially for image classification  (Basaia et al., 2019). Recently, deep learning algorithms have rapidly evolved and more complex convolutional neural network (CNN) architectures were developed such as AlexNet (Krizhevsky, Sutskever & Hinton, 2012), GoogleNet (Szegedy et al., 2015), VGGNet (Simonyan & Zisserman, 2014) and ResNet (He et al., 2016; Szegedy et al., 2017). Different from the convention sequential network architectures like AlexNet and VGG, ResNet utilizes a set of building blocks to construct the network and demonstrates the ability to greatly improve the depth of the network while having fast convergence. The applications of CNN in MRI classification, which utilize MR images and brain states labels as input and are trained end-to-end, can achieve great performance with over 90% accuracy (Cheng et al., 2017; Hosseini-Asl, Keynton & El-Baz, 2016; Jo, Nho & Saykin, 2019; Korolev et al., 2017; Lin et al., 2018; Liu et al., 2018). On the other hand, the deep neural network model, often regarded as a black-box, lacks transparency and cannot be interpreted directly. However, understanding how an MRI classification model work can help us to infer the disease regions for biomarker and therapeutic targets identification.

In this study, to address the limitations in current AD classification, we built as well as systematically compared three classification models based on SVM and two deep learning algorithms, 3D-VGGNet and 3D-ResNet, respectively, for AD prediction and disease region identification. In our SVM classifier, instead of extracting the features from ROIs, the voxel-wise intensity features were extracted after preprocessing MR images. A mask generated from the raw images was applied to perform feature selection concurrently with the classification to solve the high dimensional problem. A transfer learning strategy was adopted in the CNN models for preventing the overfitting problem and improving classification performance. The classification models were further analyzed with Gradient-weighted Class Activation Mapping (Grad-CAM) (Selvaraju et al., 2017) to revealing and visualizing respective discriminative regions, which contributed to the classification and hence potentially acted as diagnostic biomarkers for the disease.

## Methods

### The overall classification scheme

**Figure 1:**
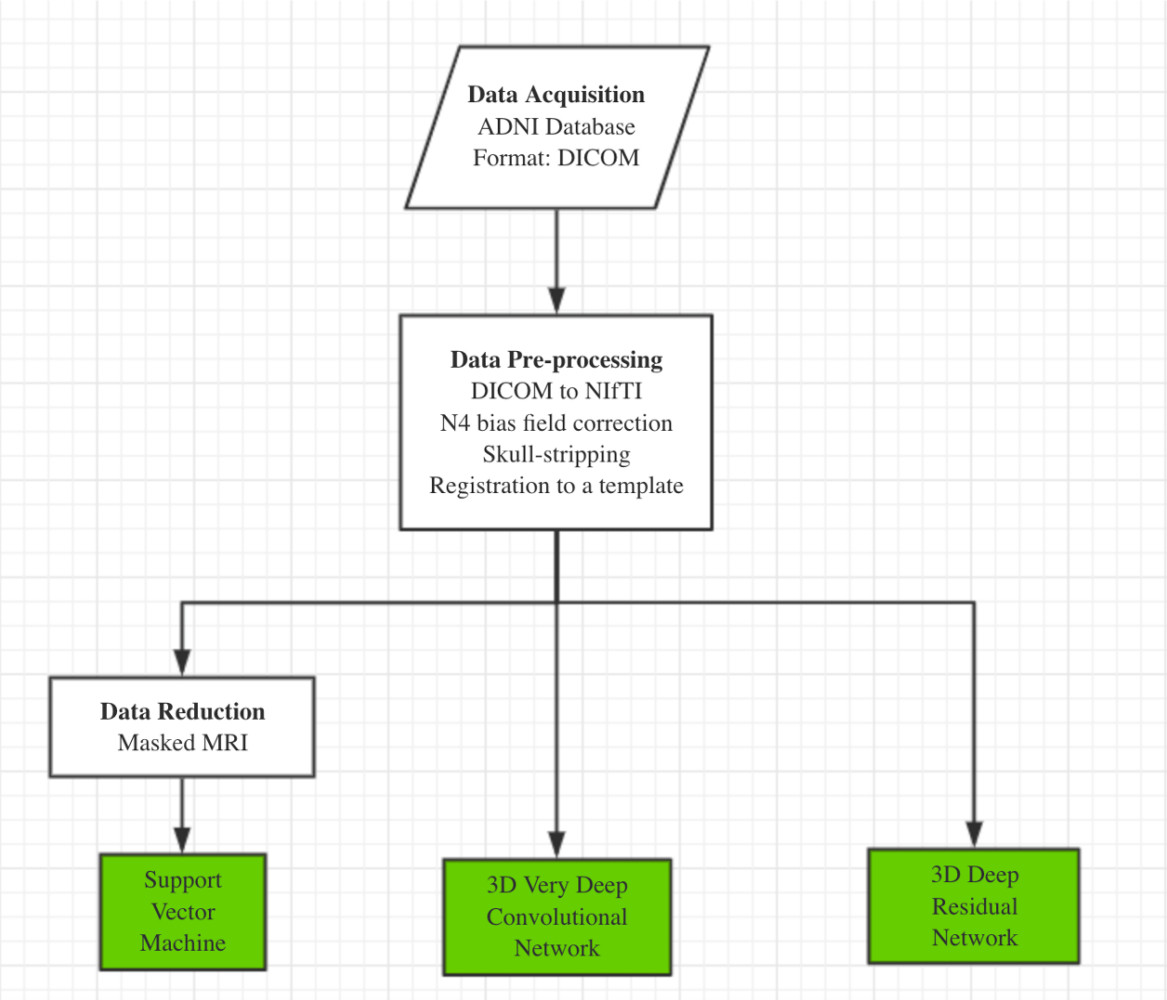
The overall workflow of our computational framework. The main procedure included data acquisition, pre-processing and classification. A traditional machine learning algorithm with feature selection and two deep 3D convolutional neural networks for Alzheimers disease classification have been investigated in this study.

Our AD classification consisted of three major steps: (i) data acquisition and pre-processing including data transformation, bias filed correction, skull-stripping, and registration; (ii) feature selection for support vector machine; (iii) deep learning algorithms with feature selection automatically. Here, two different approaches, 3D-VGGNet and 3D-ResNet, were used in the deep learning module (Fig. 1). A total of 560 MR images composed of 260 AD and 300 cognitive normal were used in the study.

### Data acquisition and preprocess

We obtained 560 T1-weighted MR images from Alzheimers Disease Neuroimaging Initiative (ADNI). The dataset included 260 AD and 300 cognitive normal (CN) older adults. All the images are in 3D (170 × 256 × 256) and acquired from 81 ADNI participants, including 42 female and 39 male subjects with an average age of 75 years old (Table S1).

The raw data of structural MRI scans for both the AD and the CN groups were provided in DICOM format in the ADNI database. The initial step was to convert DICOM format to NIfTI, as analysis on NIfTI images is more convenient than on DICOM. After that, each individual NIfTI images was corrected to uniform intensity based on the N4 bias field correction algorithm (Tustison et al., 2010). All non-brain tissues were removed from the data. Then, we registered the images to the MNI template (MNI152 T1 one mm brain) (Grabner, Neubauer & Stern, 2006) by a 12-parameter affine transformation using the FLSR package. Image registration aligned all images into one coordinate system, which is necessary for comparing or integrating the data sets. After registration to the MNI template, the dimension of the 3D volume was 182 × 218 × 182.

### SVM based model

Each MRI volume was treated as a feature vector in a high-dimensional space, where each feature was the MRI signal at a specific voxel. We applied the SVM algorithm to map whole MRI volumes from different subjects to brain states without prior selection of features. In an MRI scan, an image volume often contains thousands of voxels, resulting in a very large spatial dimension of MRI data. Training an SVM classifier for feature vectors with such high dimensions is quite computationally intensive. Therefore, data reduction is important. A mask generated from the raw images was used to remove the out of brain voxels to enhance the performance of the classifier. Dimension was reduced by selecting 10% relevant voxels using ANOVA—an F-score based feature selection (https://nilearn.github.io/decoding/decoding_intro.html). The linear kernel was adopted in the SVM classifier. Cross-validation was used for performance evaluation.

### Network architecture

The 3D-VGGNet (Simonyan & Zisserman, 2014) we used contained four blocks of 3D convolutional layers and 3D max pooling layers, followed by a fully connected layer, a batch normalization layer, a dropout layer, another fully connected layer, and the softmax output layer to produce the probabilities of AD and CN. More details of the 3D-VGGNet model architecture were presented in Fig. S1. Batch sizes of 5 were assigned to 3D-VGGNet to cope with the limited GPU memory. The deep networks were stable after iteration of about 50 epochs. Adam (Dauphin, De Vries & Bengio, 2015) with a 2.7*e*^−5^ learning rate was used as the optimizing function. The small learning rate was used here to avoid overshooting the minimum. The categorical cross-entropy was adopted as the model loss function.

The 3D-ResNet model was composed of six residual blocks (Fig. S2). Each residual block consisted of two 3D convolutional layers with 3 × 3 × 3 filters that have a batch normalization layer and a rectified- linear-unit nonlinearity layer (ReLU) between them. Each residual block fed the output of two convolutional layers and one ReLU layers and also bypassed the input to the following layers. Skip connections (identity mapping of the input) added a residual block element-by-element to the following residual block, explicitly allowing the following block to learn a residual mapping instead of a full mapping. For optimization, Nesterovs accelerated stochastic gradient descent (Nesterov, 2003) was used. Optimization parameters were set as a 2.7*e*^−5^ for learning rate with Adam optimizer, four for batch size, and 80 for training epochs. The same loss function as 3D-VGGNet and the two-class cross-entropy function were employed. The final fully connected layer of 3D-ResNet yielded the probabilities of AD and CN with softmax transformation. More details of the 3D-ResNet model architecture were presented in Fig. S2.

### Transfer learning

We applied a transfer learning strategy to prevent overfitting problem in the training of the deep learning models. The weights of a pre-trained model were transferred and adopted as the initial weights of our models. The previously trained model was chosen from (Yang, Rangarajan & Ranka, 2018). We leveraged knowledge from the pre-trained source model with the features learned from our training data to construct the AD classification model. A small learning rate was adopted for reducing the risk of losing previous knowledge. Transferring the learned features significantly reduced training time and increased network efficiency.

### Mapping disease regions

We used a Grad-CAM method (Selvaraju et al., 2017), highlighting brain parts that were essential for Alzheimers disease classification, to obtain visual explanations. Grad-CAM utilizes gradient information of the target class, which flows into the last convolutional layer and contains the spatial information indicating discriminative regions for classifications. The results showed the coarse locations of image discriminative features that were used by the model for classification.

### Environment set up for deep learning algorithms

The scripts for implementing the deep learning algorithm were written in Python 3.5. All software and library dependencies bundled were packaged in a Singularity container (Kurtzer, Sochat & Bauer, 2017), ensuring quick and easy reproducibility of the results. The two neural networks were implemented in the Lasagne library with Theano (Bastien et al., 2012) as the GPU backend support. The experiments were conducted on a 12GB Nvidia Tesla GPU card.

## Results

### The performance of SVM based model

**Figure 2:**
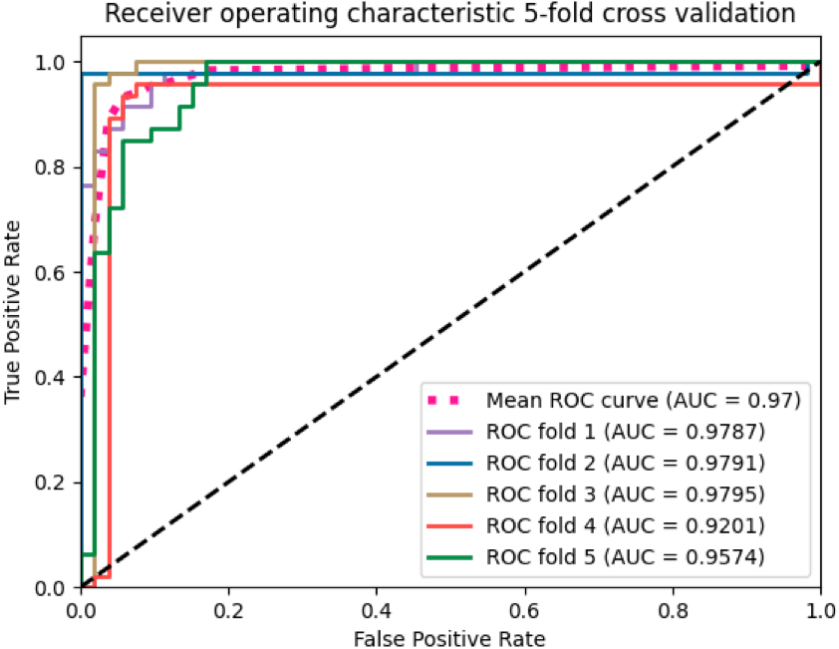
The ROC curves of the SVM classifier. The curves were generated based on the true positive rate (*y*-axis) vs. the false positive rate (*X*-axis). The area under the curve (AUC) represented the efficacy of the method.

We applied the SVM algorithm to map whole MRI volumes from different subjects to brain states without prior selection of features. As each image contains many thousands of voxels, to reduce the computational cost, a mask generated from the raw images was applied to remove the voxels outside of the brain. Then, the features obtained from the ANOVA feature selector were used as inputs to a linear kernel SVM classifier. We used 500 images for training and the remaining 60 for testing. The model achieved around 90% classification accuracy with a sensitivity of 93.94% and specificity of 85.19%. We employed stratified 5-fold validation to evaluate the robustness of the classifier. The resulting mean value of AUC (area under curve) was 0.97 (Fig. 2).

**Figure 3:**
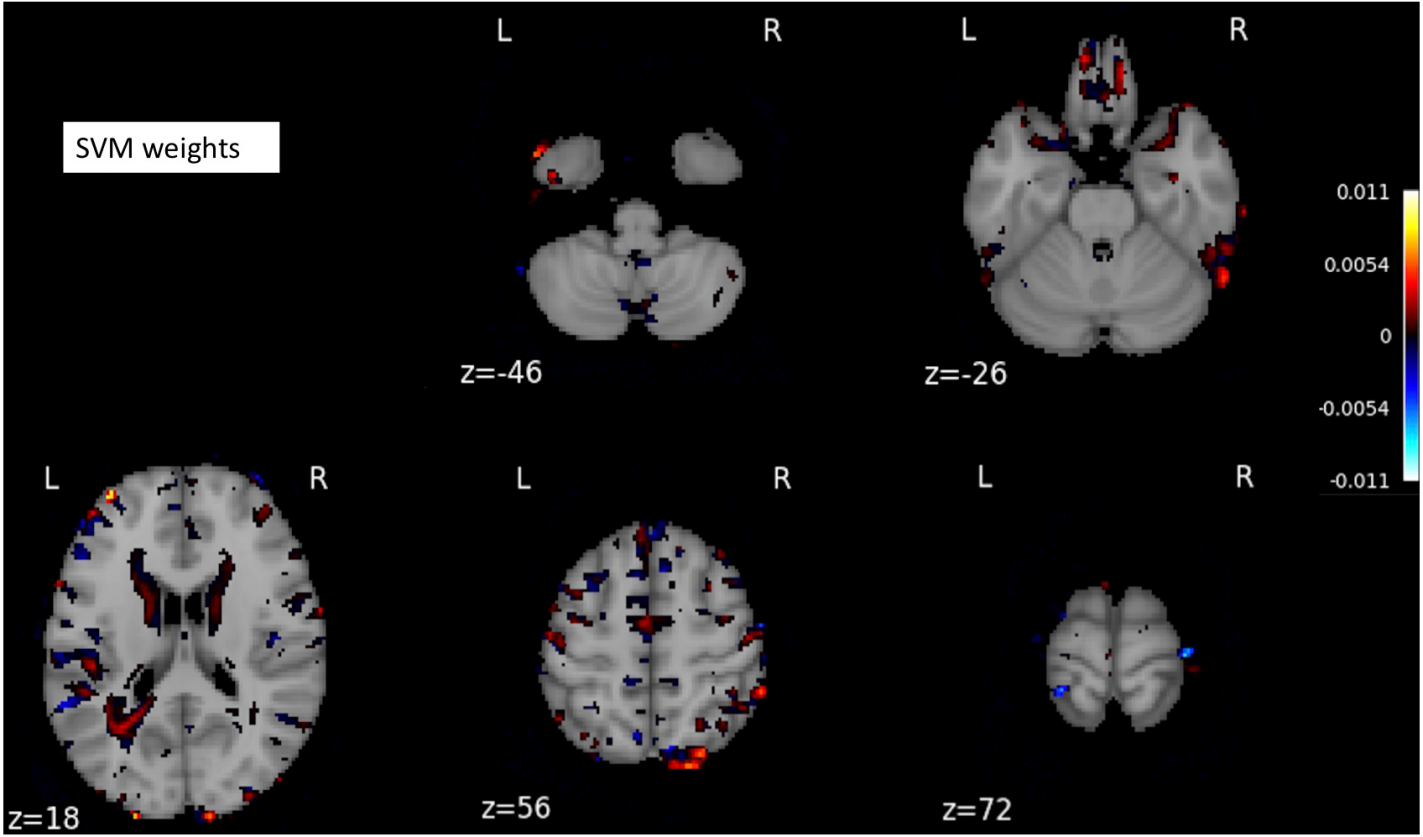
A structure scan in axial slices at different *z* levels. Projecting the weight vector back to an MR image yielded a map of the most discriminating regions between AD and CN (cognition normal). The highlighted red and blue regions represent the most discriminating regions in the original space, according to the SVM model learned from our dataset. The size of the MNI template used in visualization was 182 × 218 × 182.

The SVM classifier returned a weight vector, representing the importance of individual features for separating distinct cognitive states. As we used the reduced representation of data as input to the SVM classifier, we essentially obtained the weight vector for the reduced representation. To recover the volume with the most discriminating regions in the original space, we mapped the weight vector back to the high-dimensional space. As a result, we obtained a discriminative map, showing the brain regions that contributed most to AD classification (Fig. 3).

### The performance of deep learning-based models

The MR images were split into training and testing datasets, containing 500 and 60 images, respectively. Then, through random shuffling, 80% of training data were assigned for actual training and the remaining images for validation. The validation set was used for model optimization in the training process. The testing dataset was the same for 3D-VGGNet and ResNet models, and it was independent of training and validation sets. The voxel-wise features of one MRI sample were of high dimensionality while our sample size was limited. Hence, there were far more features than the training subjects, which can lead to an overfitting problem and poor performance. We employed techniques including batch normalization, dropout, and cross-validation in our deep network to prevent overfitting.

We first construct the classification models from scratch by randomly initializing model parameters. During the training of the 3D-VGGNet model, we found that the training loss was decreasing whereas the validation loss increased over time (Fig. S3), suggesting the overfitting. The 3D-ResNet model was failed to build from the scratch as the required memory exceeded our GPU memory capacity even after the batch size was adjusted to two (Table S2).

Thus, we applied a transfer learning strategy for model construction. The weights of a pre-trained model were transferred and adopted (Yang, Rangarajan & Ranka, 2018) as the initial weights of our models. Compared to the model trained from scratch, we found that utilizing transfer learning increased classification accuracy while reduced the training convergence time. The validation accuracy and AUC of 3D-VGGNet were increased from 0.759 to 0.97 and 0.74 to 0.994, respectively (Table S2). The training time per epoch reduced from 788s to 123s (Table S2). Collectively, our results suggested that transferring learning significantly reduced the training time and boosted efficiency of the network. The models that were trained using transfer learning were utilized in our classification and the subsequent analysis.

**Figure 4:**
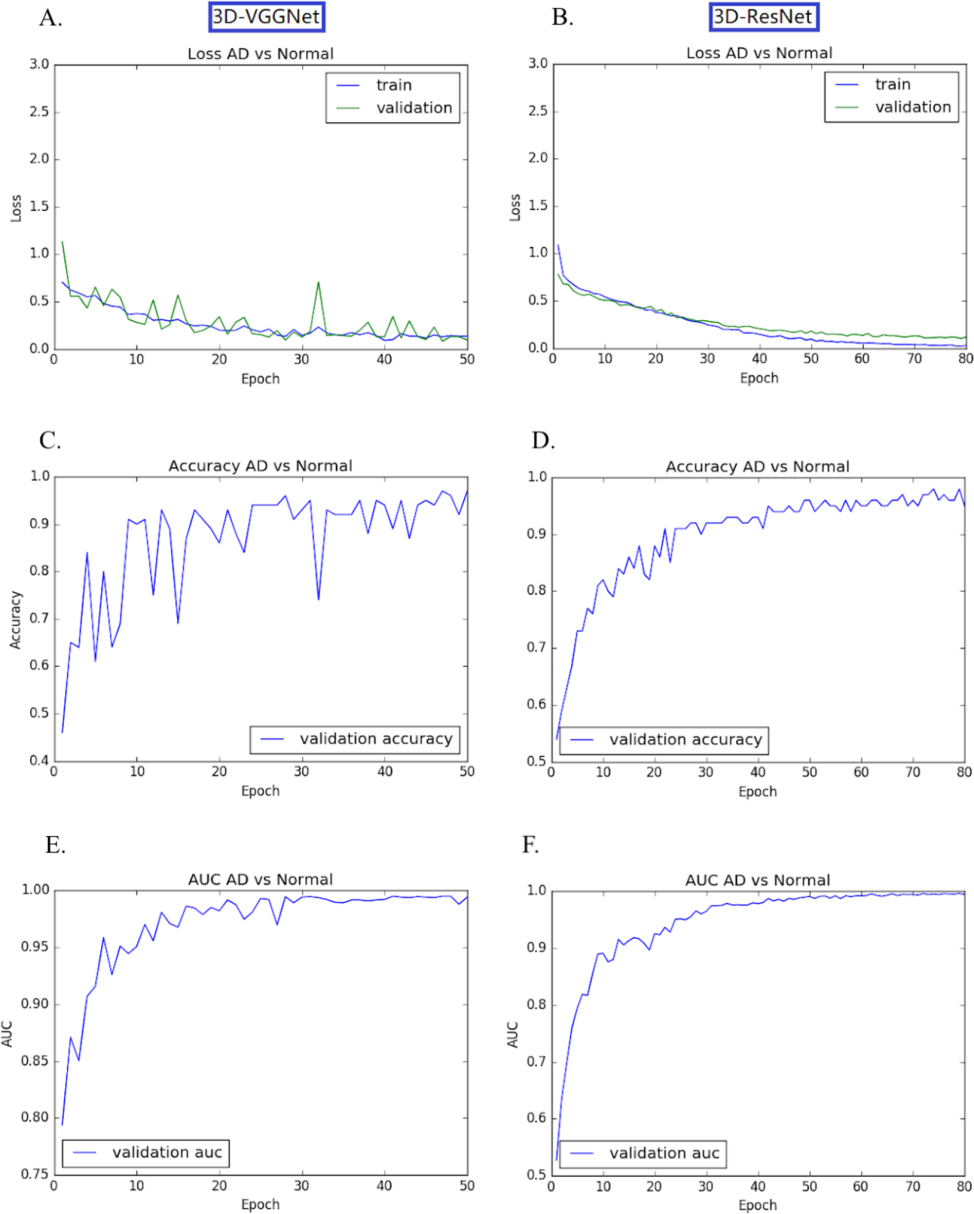
Training and validation process of 3D-VGGNet (A, C, E) and 3D-ResNet (B, D, F). The epoch numbers for VGGNet and ResNet were 50 and 80, respectively. (A, B): Training and validation loss plot; (C, D): Validation accuracy plot; (E, F): AUC plot. In the training process, the performance 3D-VGGNet and 3D-ResNet classifiers constantly improved and then plateaued after the 50th epoch and 80th epoch, respectively.

The transfer learning-based modes achieved high accuracy without the overfitting problem. Both training loss and validation loss decreased over the training epochs (Figs. 4A, 4B). In the training process, the performance 3D-VGGNet and 3D-ResNet classifiers constantly improved and then plateaued after the 50th epoch and 80th epoch, respectively (Figs. 4C–4F). We applied these two models to the 60 testing images and both classifiers achieved 95% accuracy and similar AUC values, 0.994 vs 0.995 (Table 1). Compared to 3D-ResNet model, the 3D-VGGnet based model yielded higher specificity (100% vs 96%), whereas a lower sensitivity (91.4% vs 94.3%)

### Discriminative regions visualization

**Figure 5:**
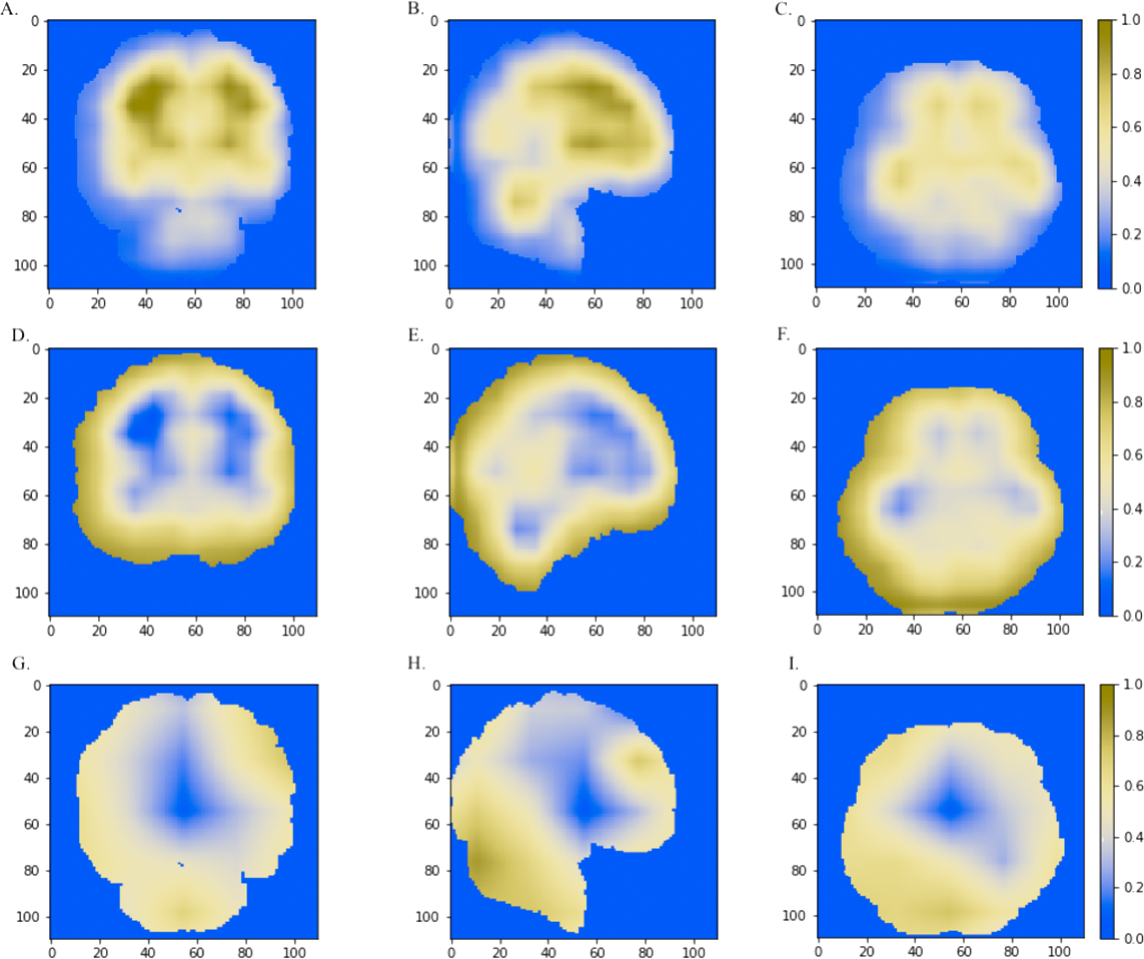
The horizontal (*x* = 50), sagittal (*y* = 50), and coronal (*z* = 50) view of the brain MRI and visual explanation heatmaps generated from 3D-ResNet (A–F) and 3D-VGGNet (G, H, I). The white matter region, the cerebral cortex and cerebellum were highlighted as shown in (A, B, C), (D, E, F) and (B, G, H, I), respectively. The size of the MNI template used in visualization was 110 × 110 × 110 and the coordinates in the other two dimensions were shown in the figure axes.

We further combined the CNN models with the Grad-CAM (Selvaraju et al., 2017) approach to visualize important areas that contributed to the classification. The last convolutional layer of the CNN contains the spatial information indicating discriminative regions for making classifications. The white matter region was highlighted in normal samples (Figs. 5A–5C) as compared to AD images. Convergent neuroimaging studies have implicated micro- and macro-structural abnormalities in white matter associated with the risk and progression of AD. White matter alterations have been reported to be associated with a decline in motor function in speed and fine motor coordination (Bozzali et al., 2002; Kao et al., 2019; Nasrabady et al., 2018).

On the other hand, the cerebral cortex (the outer layer of the brain) area was highlighted (Figs. 5D–5F) in the AD image as well as in the SVM discriminative map sporadically (Fig. 3), indicating the cerebral cortex might relate to Alzheimers disease. Pathologically, Alzheimers disease is associated with neuronal death and gliosis specifically in the cerebral cortex (Li et al., 2018). Anatomical properties, such as volume, gray matter density, and thickness of the cerebral cortex have been used to identify AD in various ROI-based MRI quantitative analyses. Those studies have shown that thickness was reduced in regions of the cerebral cortex that were affected pathologically in AD (Bakkour, Morris & Dickerson, 2009; Dickerson et al., 2009; Lerch et al., 2005).

Interestingly, the cerebellum was highlighted in the maps generated based on 3D-VGGNet model (Figs. 5G– 5I) and in the one based on 3D-ResNet model (Fig. 5B). The cerebellum communicates with the cerebral cortex and is responsible for emotion and cognition, however, the cerebellum is a relatively neglected area of the AD brain and is not commonly be considered involved in the pathophysiology of Alzheimers disease (Jacobs et al., 2018). The spatial information we learned from our models suggested that the cerebellum may have roles in the clinical phenomenology of the disease.

**Table 1:** Comparison of three different methods for AD prediction.

**Classifier**	**Accuracy**	**Sensitivity**	**Specificity**	**AUC**
SVM	0.90	0.939	0.851	0.97
3D-VGGNet	0.95	0.914	1	0.994
3D-ResNet	0.95	0.943	0.96	0.995

### Classification model comparison

A traditional machine learning algorithm and two deep 3D convolutional neural networks for Alzheimers disease classification were investigated in this study. Among them, the SVM classifier had the lowest accuracy (90%), and two CNN classifiers achieved the same accuracy of 95% on our testing dataset (Table 1). VGGNet is a common feed-forward network with convolutional and pooling layers, while ResNet is a modern residual neural network. The skip connections can facilitate the learning process for deeper networks in the ResNet model. Our 3D-ResNet model yielded higher sensitivity and AUC value than the 3D-VGGNet (Table 1) classifier.

**Figure 6:**
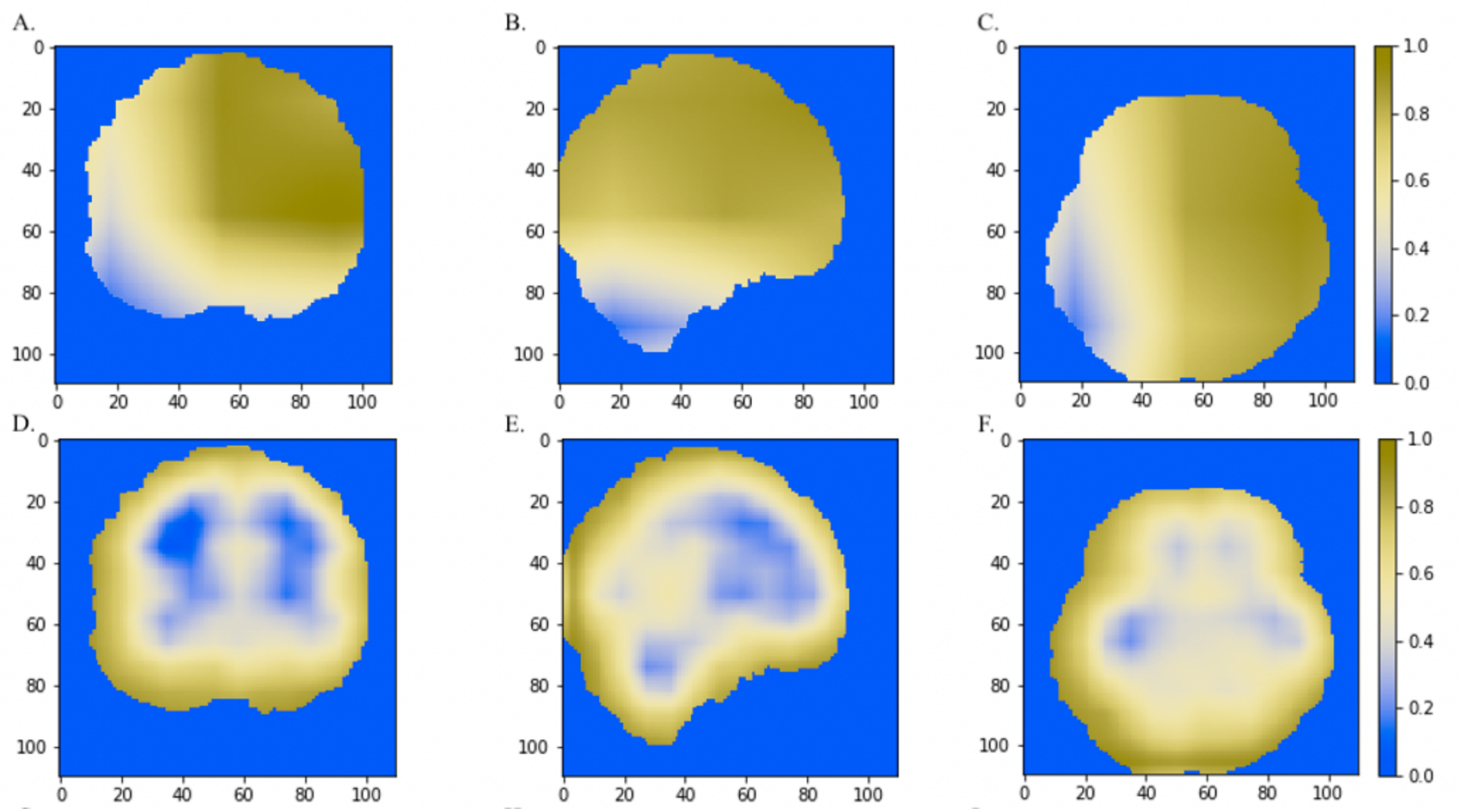
The comparisons of discriminative regions heatmaps in horizontal (*x* = 50), sagittal (*y* = 50), and coronal (*z* = 50) views of the brain MRI. The heatmaps generated by Grad-CAM based on 3D-ResNet were shown in A, B and C, whereas the ones based on 3D-VGGNet model were shown in D, E and F. The size of the MNI template used in visualization was 110 × 110 × 110 and the coordinates in the other two dimensions were shown in the figure axes.

We also compared the heatmaps generated by Grad-CAM based on the convolutional layer activation of the VGGNet and ResNet models. In the discriminative maps, the brain regions that were essential for classification were highlighted. We found that Res-3D-Grad-CAM generated the maps with higher resolution and accuracy than VGG-3D-Grad-CAM (Figs. 6D, 6E, 6F vs 6A, 6B, 6C). The Grad-CAM approach has a low-resolution problem and it produces a coarse heatmap of the same size as the last convolutional layer. The size of the last convolutional layers of our 3D-VGGNet and 3D-ResNet was 3 × 3 × 3 and 14 × 14 × 14, respectively. The smaller size of the convolutional layer of 3D-VGGNet model may result in loss of details and lower localization accuracy.

## Discussion

Alzheimers disease currently lacks effective treatment. The computation classification model contributes to early diagnosis as well as better monitor and treatment plans. In this study, we constructed and compared three classification models using SVM, a traditional machine learning algorithm and two deep learning algorithms, 3D-VGGNet and 3D-ResNet, respectively. Overall, deep learning models outperformed the SVM model. On the other hand, deep learning models are computationally expensive and memory intensive.

Handcraft feature selection is needed for traditional machine learning approaches such as SVM. The ROI-based feature selection for MR images substantially reduced the feature dimension and offered robust representation for classification. However, this type of method relies on prior hypothesis and tiny abnormal alterations can be overlooked. We hence used voxel-wise intensity as input features and apply a mask and ANOVA to reduce feature dimensionality. Our SVM classifier achieved comparable accuracy as the other SVM classifiers (Lama et al., 2017; Ledig et al., 2018). However, discriminative patterns were highlighted sporadically, and we could not track a disease-associated brain region. It has been reported that that patterns of brain image intensity and atrophy in older adults (age > 75) are very similar (Sarraf, Tofighi & Initiative, 2016; Vemuri, Jones & Jack, 2012). Manually selecting features for separating AD from healthy subjects could be challenging.

The 3D-VGGNet and 3D-ResNet models automatically learn the latent features of the raw data, incorporating feature selection in the process of classification. Both CNN models trained using a transfer learning technique achieved a prediction accuracy of 95%. We found that with a limited number of samples, a total of 560 MR images in our study, building models from scratch was not efficient compared to the developing models using transfer learning strategy. We were unable to build a 3D-ResNet model from the scratch due to the out of memory issue, while the accuracy of the 3D-VGG model constructed from the scratch yielded accuracy around 75% which was significantly lower than the accuracy 95% yielded by the classifier built based on transfer learning. Additionally, adopting transfer learning substantially decreased training time. When there are no sufficient samples for optimizing the hyperparameters, the deep learning model development can substantially benefit from transfer learning.

To visualize and identify the brain regions that contribute to classification, we projected the weighted feature vector back to the original image for the SVM classifier, and utilized Grad-CAM to highlight the brain parts essential for AD classification for the two CNN models. The cerebral cortex and cerebellum were consistently highlighted in the resulting maps. The cerebral cortex is a well-known AD-associated brain region, whereas the cerebellum is relatively neglected in the current AD study. Temporal and frontal lobe regions were reported to be affected in AD (Kumfor et al., 2014), however, they were not highlighted in our disease regions. This may attribute to heterogeneity within Alzheimers disease as our models were trained using MRI in ADNI. A model trained with large numbers of samples from diverse patient cohorts can be more effective. Advancements in computer hardware will also facilitate the development of more accurate and reliable computational models for clinical practice.

## Conclusions

Our study systematically compared three distinct classifiers for predicting Alzheimers disease and inferring the brain regions that were associated with disease development and progression. Support vector machine is a traditional machine learning method, VGGNet is a common deep neuron network and ResNet is a modern residual neural network. Our ResNet model achieved the best classification performance as well as more accurately localized the disease-associated brain regions compared to the other two approaches. The two deep learning classifiers consistently indicated the aberrant cerebellum associated with AD development.

##  Supplemental Information

10.7717/peerj.10549/supp-1 Supplemental Information 1 The details of the MRI images

10.7717/peerj.10549/supp-2 Supplemental Information 2 Comparisons the CNN models built from scratch and transfer learning

10.7717/peerj.10549/supp-3 Supplemental Information 3 The architecture of the 3D-VGGNet model

10.7717/peerj.10549/supp-4 Supplemental Information 4 The architecture of the 3D-ResNet model

10.7717/peerj.10549/supp-5 Supplemental Information 5 The training and validation losses of the 3D-VGGNet model that was constructed from scratch The blue curve represents the training loss while the green curve refers to validation loss. The gray shadows are the standard deviation of losses

## Additional Information and Declarations

### Data Availability

The following information was supplied regarding data availability: The raw data is publicly available at the Alzheimer’s Disease Neuroimaging Initiative (ADNI): http://adni.loni.usc.edu/data-samples/access-data/. To apply for access to this data: “The application process includes acceptance of the Data Use Agreement and submission of an online application form. The application must include the investigator’s institutional affiliation and the proposed uses of the ADNI data. ADNI data may not be used for commercial products or redistributed in any way.” ADNI’s data sharing policy states: “All ADNI data are shared without embargo through the LONI Image and Data Archive (IDA), a secure research data repository. Interested scientists may obtain access to ADNI imaging, clinical, genomic, and biomarker data for the purposes of scientific investigation, teaching, or planning clinical research studies. Access is contingent on adherence to the ADNI Data Use Agreement and the publications’ policies outlined in the documents listed below. Note: documents are subject to updates by ADNI.”
