# Supplementary material for: Comparison of machine learning approaches for enhancing Alzheimer’s disease classification
Source: PeerJ. 2021 Feb 25;9:e10549. doi: 10.7717/peerj.10549 (PMC7916537; doi:10.7717/peerj.10549)
Supplement: Supplemental Information 1 [file peerj-09-10549-s001.docx]

**Supp. Table 1:** The details of the MRI images.

| **Attribute** | **Value** |
| --- | --- |
| Age | 56 to 89 |
| Sex | 39 Men, 42 Females |
| Matrix Size | 170 × 256 × 256 |
| Voxel Size | 1.2 x 1 x 1 mm |
| Sequence | MPRAGE 3D T1 weighted |
| The field strength of the magnet | 3T (teslas) |
