# Supplementary material for: Comparison of machine learning approaches for enhancing Alzheimer’s disease classification
Source: PeerJ. 2021 Feb 25;9:e10549. doi: 10.7717/peerj.10549 (PMC7916537; doi:10.7717/peerj.10549)
Supplement: Supplemental Information 2 [file peerj-09-10549-s002.docx]

**Supp. Table 2.** Comparisons the CNN models built from scratch and transfer learning.

|  | **Transfer learning** | | **Learning from scratch** | |
| --- | --- | --- | --- | --- |
| **Performance measures** | 3D-VGGNet | 3D-ResNet | 3D-VGGNet | 3D-ResNet |
| Training loss | 0.135 | 0.0275 | 0.1336 | - |
| Validation loss | 0.0961 | 0.1177 | 0.8907 | - |
| Validation accuracy | 0.97 | 0.95 | 0.759 | - |
| Validation AUC | 0.9942 | 0.9946 | 0.7401 | - |
| Epoch number | 50 | 80 | 100 | - |
| Batch size | 5 | 4 | 5 | 2 |
| Runtime per epoch (Avg.) | 123s | 475s | 788s | Out of memory |
