# Supplementary figures and images for: Comparison of machine learning approaches for enhancing Alzheimer’s disease classification
Source: PeerJ. 2021 Feb 25;9:e10549. doi: 10.7717/peerj.10549 (PMC7916537; doi:10.7717/peerj.10549)

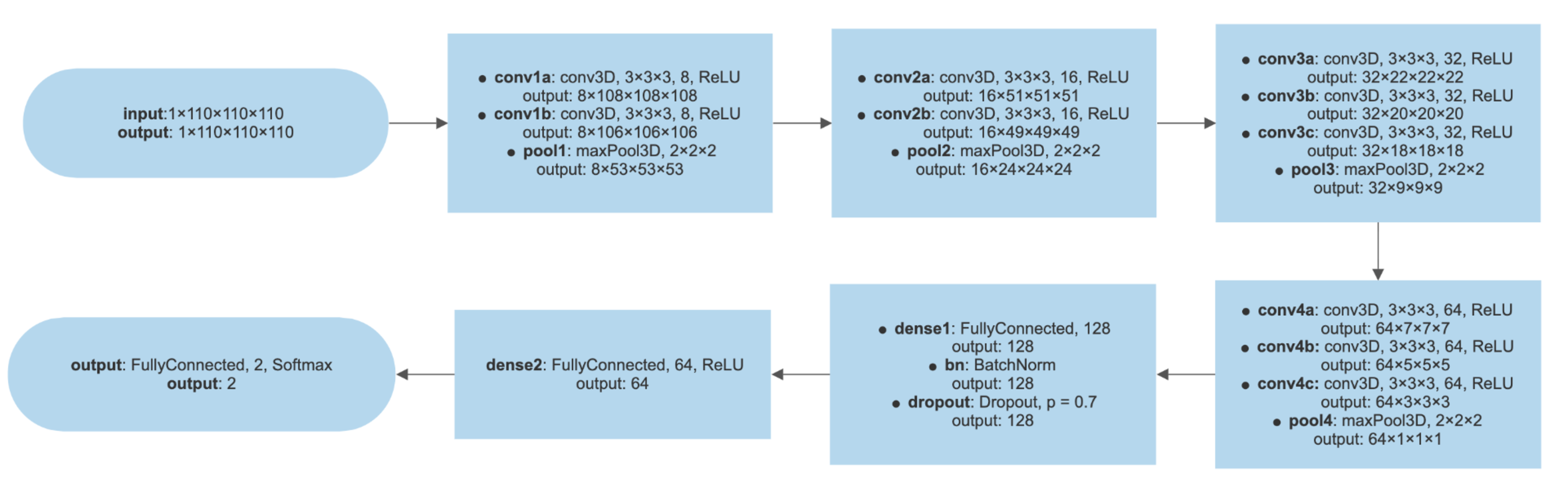

Supplement: Supplemental Information 3 [file peerj-09-10549-s003.png]

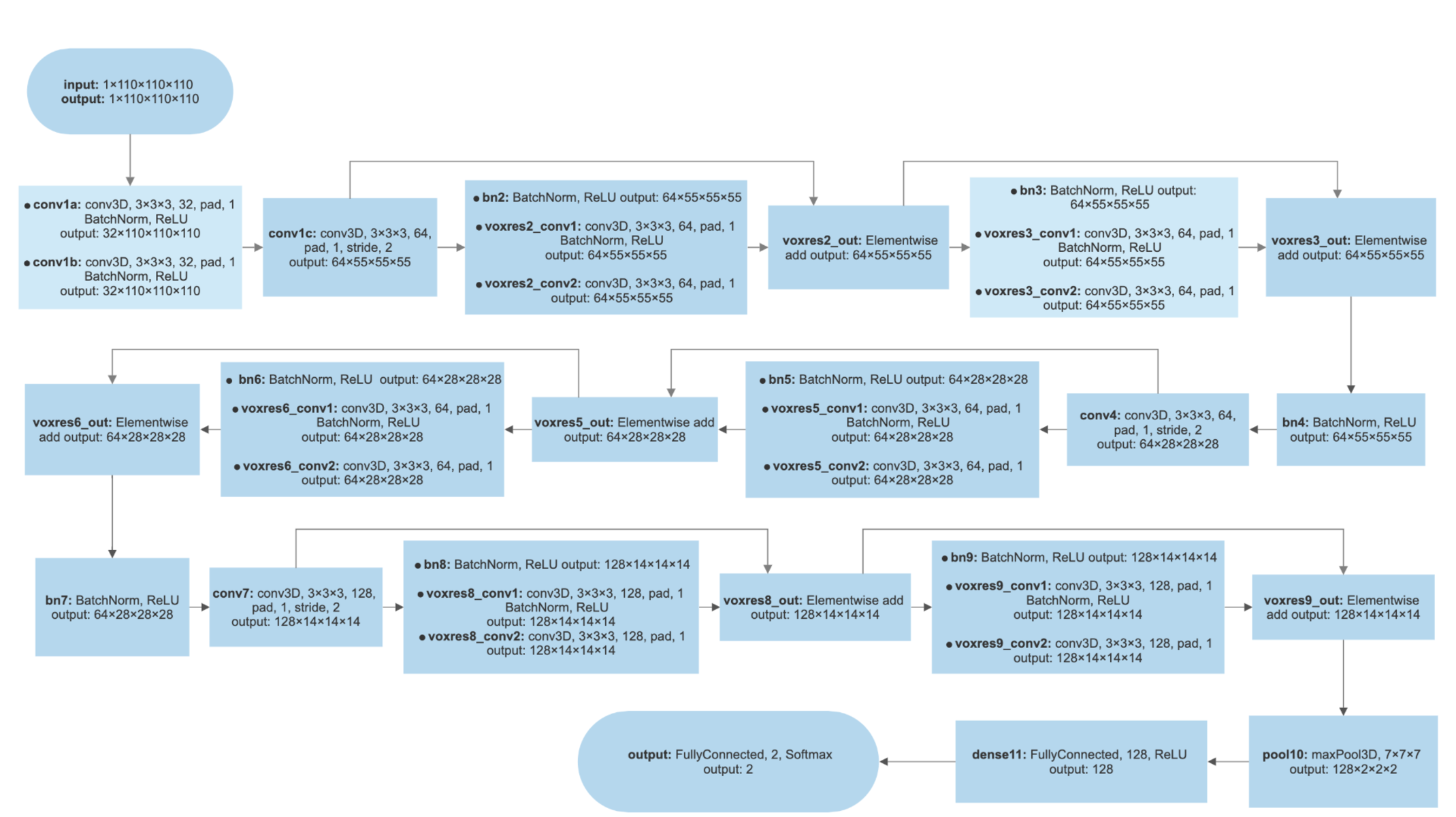

Supplement: Supplemental Information 4 [file peerj-09-10549-s004.png]

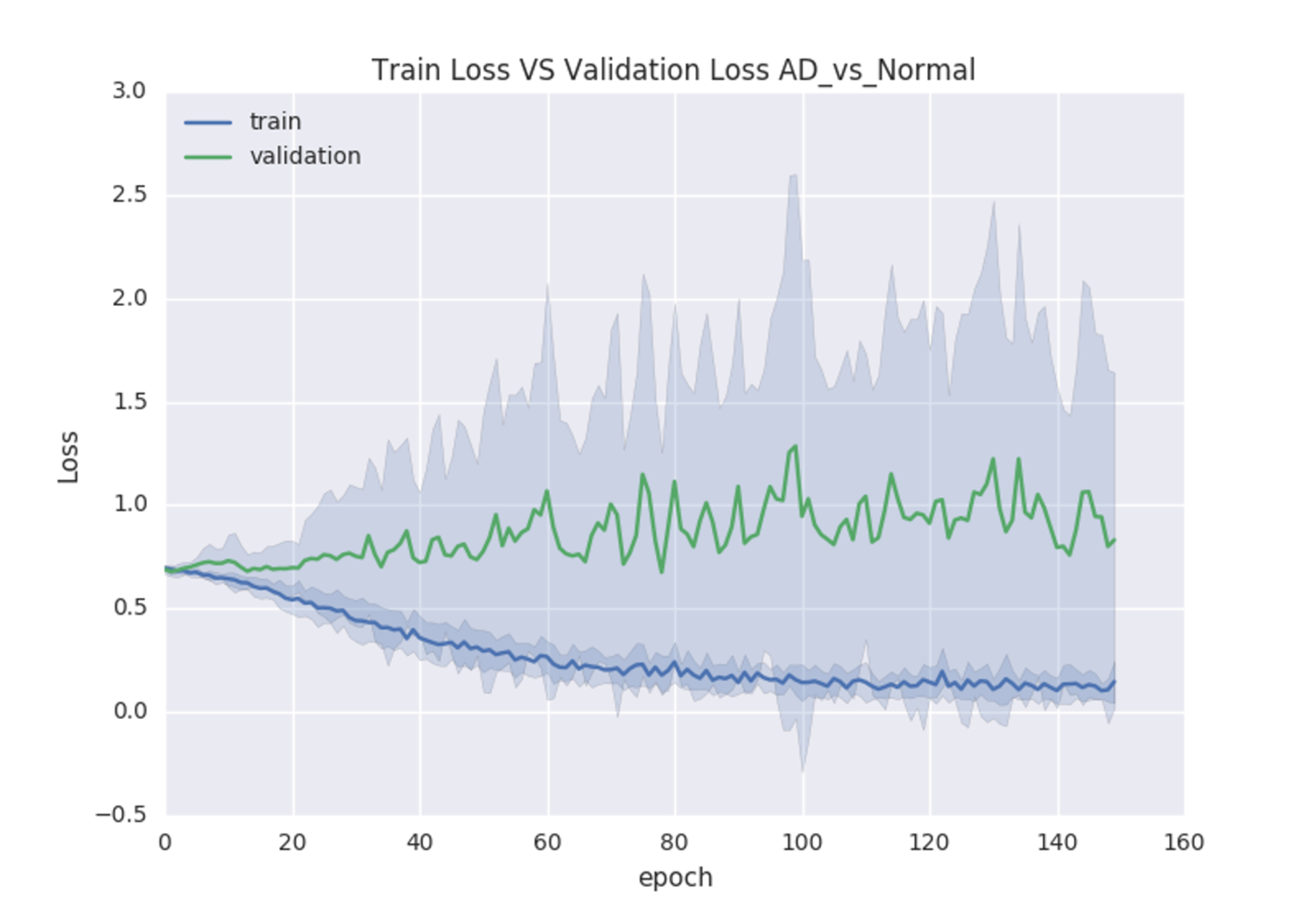

Supplement: Supplemental Information 5 — The blue curve represents the training loss while the green curve refers to validation loss. The gray shadows are the standard deviation of losses [file peerj-09-10549-s005.png]
